# Internet Searches for Terms Related to Child Maltreatment During COVID-19: Infodemiology Approach

**DOI:** 10.2196/27974

**Published:** 2021-07-13

**Authors:** Madelon M E Riem, Pietro De Carli, Jing Guo, Marian J Bakermans-Kranenburg, Marinus H van IJzendoorn, Paul Lodder

**Affiliations:** 1 Behavioural Science Institute Radboud University Nijmegen Netherlands; 2 Department of Developmental and Social Psychology Padua University Padua Italy; 3 Department of Health Policy and Management School of Public Health Peking University Peking China; 4 Clinical Child & Family Studies Faculty of Behavioural and Movement Sciences Vrije Universiteit Amsterdam Netherlands; 5 Department of Psychology Education and Child Studies Erasmus University Rotterdam Netherlands; 6 Center of Research on Psychological & Somatic Disorders Department of Medical and Clinical Psychology Tilburg University Tilburg Netherlands

**Keywords:** child, maltreatment, COVID-19, pandemic, internet searches, information-seeking, internet, abuse, trend, Google trends, infodemiology

## Abstract

We examined internet searches indicative of abusive parental behaviors before and after the World Health Organization’s declaration of COVID-19 as a pandemic (March 11, 2020) and subsequent lockdown measures in many countries worldwide. Using Google Trends, we inferred search trends between December 28, 2018, and December 27, 2020, for queries consisting of “mother,” “father,” or “parents” combined with each of the 11 maltreatment-related verbs used in the Conflict Tactics Scales, Parent-Child version. Raw search counts from the Google Trends data were estimated using Comscore. Of all 33 search terms, 28 terms showed increases in counts after the lockdowns began. These findings indicate a strong increase in internet searches relating to occurrence, causes, or consequences of emotional and physical maltreatment since the lockdowns began and call for the use of maltreatment-related queries to direct parents or children to online information and support.

## Background

With social distancing measures, school closures, and mounting unemployment rates, the COVID-19 pandemic drastically impacted the lives of families across the globe. An accumulating number of studies show that pandemic-related stressors induce mental health problems, which in turn may impede parenting abilities. For example, job loss, financial insecurities, parental anxiety, and depressive symptoms during the pandemic have been associated with increased (risk for) emotional and physical abuse [1,2]. Although these findings give rise to widespread concerns about potential increases in child maltreatment [3], data on the scope of this problem are still scarce.

A previous study on the impact of the pandemic showed that mental health problems were reflected in internet searches in the early phase of the pandemic [4]. More specifically, internet searches indicative of acute anxiety spiked in March 2020. Another study also indicated a relationship between search traffic data from Google and cases and deaths in severely affected European countries [5]. These studies motivate surveillance of such internet searches in order to track outbreaks and monitor health threats during the pandemic; hence, infodemiology (ie, information epidemiology) metrics, in particular data from Google Trends, could be useful in tracking the virus and monitoring its impact. Here, we applied an infodemiology approach to examine whether increased risk for child maltreatment during the pandemic is reflected in internet searches. We examined internet searches indicative of abusive parental behaviors (emotional and physical abuse) before and after the World Health Organization’s declaration of COVID-19 as a pandemic (March 11, 2020) and subsequent lockdown measures in many countries worldwide.

## Increases in Child Maltreatment–Related Search Terms

We used Google Trends to infer search trends between December 28, 2018, and December 27, 2020, for queries consisting of “mother,” “father,” or “parents” combined with each of the 11 maltreatment-related verbs used in the Conflict Tactics Scales, Parent-Child version [6]: yelled, screamed, shouted, cursed, swore, threatened, pinched, hit, slapped, beat, and shook. We inferred weekly raw search counts from the Google Trends data using the R package *gtrendR* [7]. This package first obtains global relative search trends for a particular search term (using quotes) from the Google Trends database. It then uses Comscore estimates to retrieve the total number of weekly Google desktop searches within a particular time period. For our analysis, this number was 3044 million searches per week for desktop searches and 6088 million searches in total, assuming that desktop searches amount to 50% of total Google searches. We fitted a seasonal autoregressive integrated moving average model using the R package *gtrendsr* [8] to estimate the excess search count after the start of the pandemic compared to the years before, while taking into account seasonal patterns recurring throughout the year. The increase in search activity was estimated by taking the ratio of the average weekly number of searches before and after the interruption point (March 11, 2020, when the World Health Organization declared COVID-19 a pandemic). All analyses were conducted using R statistical software (version 3.5.0; R Foundation for Statistical Computing) and the script can be found on the Open Science Framework [9].

**Figure 1:**
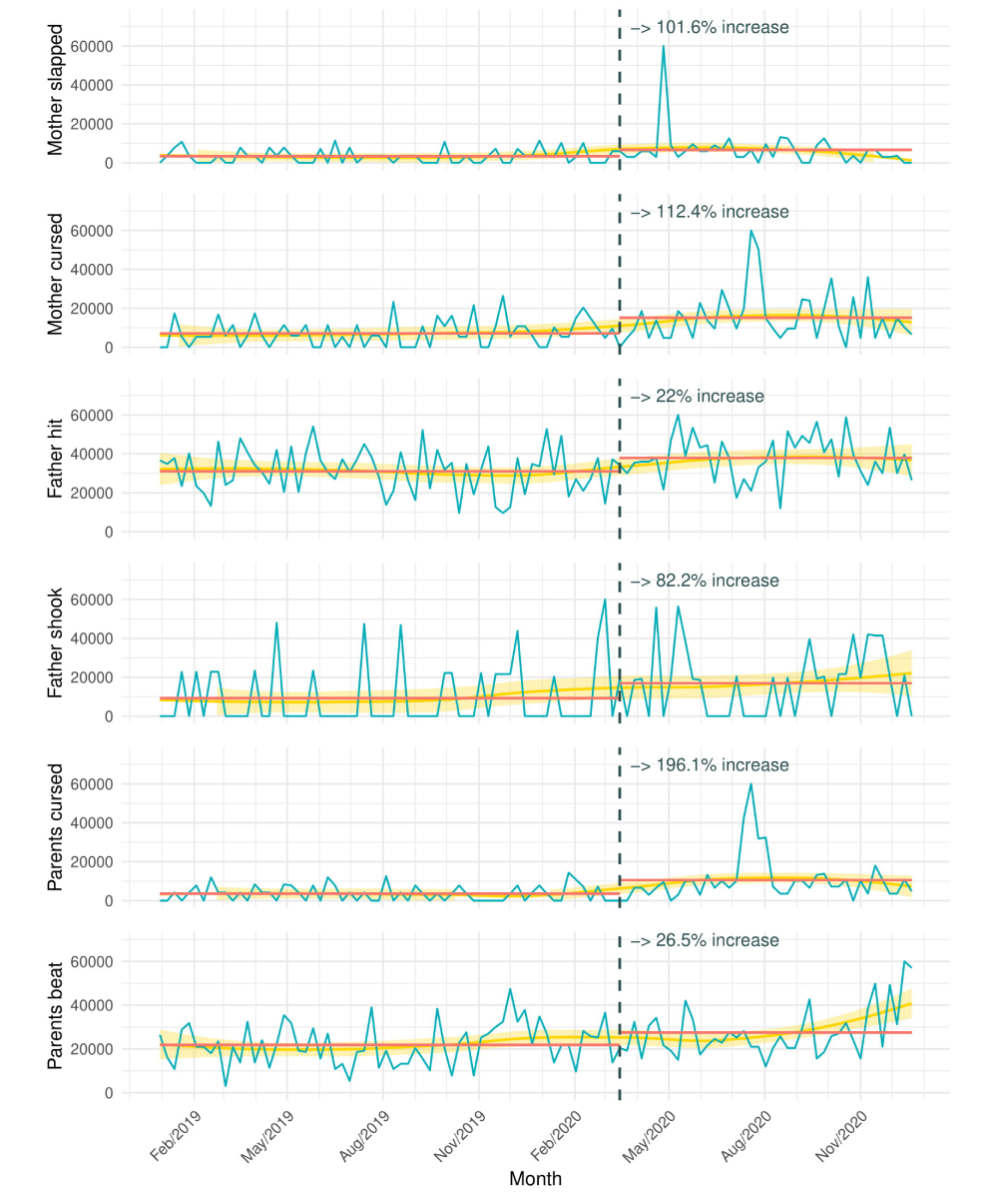
Google search trends for abuse-related verbs. The blue solid lines indicate the raw estimated weekly search counts and the gold shaded lines indicate the fitted LOESS regression curve including a 95% CI. The red lines represent the average estimated search counts before and after the World Health Organization's declaration of COVID-19 as a pandemic (black dotted line). Differences in raw search counts should only be interpreted within (and not between) search terms. For more details, see the R script available on the Open Science Framework site [9].

Figure 1 visualizes search trends before and after lockdowns began for the terms showing a pronounced increase: “mother slapped,” “mother cursed,” “father beat,” “father shook,” “parents cursed,” and “parents beat.” Several other search terms showed similar increases (see [9]). Of all 33 search terms, 28 showed increases in counts (range: 1.8%-196.1%; Table 1).

**Table 1:** Percentages indicating increases in search terms since the COVID-19 lockdown started.

Terms			Father, %		Mother, %		Parents, %
**Psychological abuse**							
	Yelled	48.6		13.2		48.6	
	Screamed	51.2		57.4		—^a^	
	Shouted	87.0		54.6		23.6	
	Cursed	42.2		112.4		196.1	
	Swore	15.0		32.7		—	
	Threatened	29.0		28		82.3	
	Average change	45.5		49.7		87.7	
**Physical abuse**							
	Pinched	—		81.8		—	
	Hit	22.0		1.8		7.2	
	Slapped	17.9		101.6		4.6	
	Beat	—		7.7		26.5	
	Shook	82.2		51.9		45.5	
	Average change	40.7		49.0		21.0	

^a^Not available; search terms without percentages did not yield sufficient data.

## Conclusions

These findings indicate a strong increase in internet searches relating to occurrence, causes, or consequences of harsh parenting since the lockdowns began. We cannot determine who is searching (eg, parents, family members, children, others) and whether searches are directly linked to specific experiences, witnessing maltreatment, or acts of emotional and physical maltreatment. However, our findings suggest an increased risk for child maltreatment due to the COVID-19 pandemic and may motivate the development of novel data-driven family support strategies. Moreover, our findings extend previous studies showing that an infodemiology approach could be an integral part of the surveillance of the pandemic and its impact [4,5]. This topic warrants further research to examine how increases in maltreatment-related search queries are related to actual regional increases in prevalence of maltreatment during the pandemic.

During the pandemic, child maltreatment has progressed from unnoticed to invisible due to social distancing [10]. Observing maltreatment-related search activity may be a way to monitor increases in child maltreatment and inform policy makers to stimulate (preventive) intervention. Importantly, internet searches can also be used to target support to families at risk during pandemics. With the “OneBox” approach, Google refers searchers using suicide-related queries to links to local helplines that are highlighted at the top of search results. Maltreatment-related Google queries, however, do not result in any helpline referrals. As already implemented for search terms like suicide, it is time for maltreatment-related queries to direct parents or children to online information and support.
